# Challenging oneself on the threshold to the world of research – frail older people’s experiences of involvement in research

**DOI:** 10.1186/s12877-020-01817-z

**Published:** 2020-10-17

**Authors:** Isak Berge, Emmelie Barenfeld, Synneve Dahlin-Ivanoff, Maria Haak, Qarin Lood

**Affiliations:** 1 Department of Health and Rehabilitation, Institute of Neuroscience and Physiology, Sahlgrenska Academy, Centre for Ageing and Health – AgeCap, University of Gothenburg, Gothenburg, Sweden; 2 Institute of Health and Care Sciences, Sahlgrenska Academy, University of Gothenburg, Gothenburg, Sweden; 3 University of Gothenburg Centre for Person-Centred Care (GPCC), Sahlgrenska Academy, University of Gothenburg, Gothenburg, Sweden; 4 Department of Psychiatry and Neurochemistry, Institute of Neuroscience and Physiology, Sahlgrenska Academy, Centre for Ageing and Health – AgeCap, University of Gothenburg, Gothenburg, Sweden; 5 Research Platform for Collaboration for Health, Faculty of Health Science, Kristianstad University, Kristianstad, Sweden; 6 Department of Health Sciences, Faculty of Medicine, Lund University, Lund, Sweden; 7 School of Nursing and Midwifery, La Trobe University, Bundoora, Australia

**Keywords:** User involvement, Frailty, Person centred, Grounded theory, Ageing, Patient and public involvement, Research participation

## Abstract

**Background:**

User involvement of people outside academia in research is argued to increase relevance of research for society and to empower the involved lay persons. Frail older people can be a hard to reach group for research and thus an underrepresented group in research. There is a lack of knowledge how collaboration with frail older people should be best performed. Therefore, the aim of this study was to explore frail older people’s experiences of involvement in research.

**Methods:**

In this study we have invited people, 75 years of age or older screened as physically frail and who have previously participated in a study as data sources, to share their experiences by intensive interviewing. Data was collected and analysed in parallel inspired by a constructivist grounded theory approach.

**Results:**

The results demonstrate how frail older people have different incentives, how their context of ageing and the unusual position of being involved in research altogether influenced how, where and in what way they wished to be involved in research. This is described in three categories: *Contributing to making a difference for oneself and others, Living a frail existence* and *Being on somebody else’s turf.* The categories compose the core category, *Challenging oneself on the threshold to the world of research*, which symbolises the perceived distance between the frail older people themselves and the research world, but also the challenges the frail older people could go through when choosing to be involved in research.

**Conclusions:**

Frail older people have a varied capacity to participate in research, but in what way and how is difficult to know before they have been involved in the process of research. Our results advocate that it is problematic to exclude frail older people a priori and that there is a potential for new perspectives and knowledge to be shaped in the encounter and in the relationship between the researcher and the frail older person. For research to be able to cater for frail older people’s needs of health services, their voices need to be heard and taken into consideration.

## Background

There are several different reasons as to why researchers may choose to involve people outside of academia in research projects. One reason is to have people outside academia to influence the design and conduct of the study and thus increase the relevance of research findings for society as a whole and to empower lay persons when collaborating with researchers [1]. Another reason is due to the problem of underrepresentation of very old or frail older people in research. However, there is a lack of knowledge and structure for how and when research could be conducted involving frail older people. Our previous research has intended to involve the target group of frail older people in the planning and implementation of research, but involving and collaborating with frail older people has not always been easy [2, 3]. Due to their health status as frail, they can be a hard-to-reach group [4]. Barriers for user involvement could be their degree of frailty, morbidity and disability.

One reason for frail older people’s underrepresentation in health research could be that they are often directly excluded from participation, for example in randomised controlled trials. This exclusion could be based on notions that they would not be able to participate, or that the results would not be beneficial to them [5]. In a review, Thake & Lowry (2017) describe that 92.8% of the investigated clinical trials that had a specified upper age limit did not include any justification of why this was the case [6]. As frail older people often have several illnesses, they can also be excluded indirectly from research in the quest for a ‘clean sample’ in the population studied [5]. An example of indirect exclusion is how frail older people are excluded by the fact that it is a common requirement for participation in research to have cognitive abilities intact [7]. Therefore, frail older people’s underrepresentation runs the risk of results from health research being irrelevant for them and that it might be difficult to organise healthcare that will be able to meet frail older people’s needs.

User involvement in research is a way of involving lay people in research and thereby make it more relevant and closer to the group of users that the research aims to benefit [8]. The users can be involved in one or more of the different parts of the research process, such as contributing to the determination of research questions, project applications, data collection, analysis, compilation and dissemination of results. There is a difference between user involvement and participation in more conventional research as data sources [1]. In contrast to participating as data sources, user involvement in research provides a possibility for new knowledge spaces to be created, where knowledge can be co-created in collaboration between researchers and lay persons [9]. One consequence of the direct and indirect exclusion of frail older people is that there is no scientific evidence on how research in collaboration with frail older people should be performed. Therefore, in order to fill the current knowledge gap about how involvement of frail older people in research could be optimised, the aim of this study was to explore frail older people’s experiences of involvement in research.

## Method

### Design

A qualitative design inspired by Charmaz’s [10] constructivist grounded theory was chosen for the study. This is a method suitable for studying processes and exploring actions in their context, where research and data are constructed in the interactions between researchers and participants. Constructivist grounded theory contributes with understanding rather than trying to explain the process that is studied [10]. The method was chosen in order to be able to give an increased understanding of frail older people’s involvement in research based on their thoughts and experiences.

### Participants

To be able to explore frail older people’s experiences of participating in research, participants were recruited among people 75 years of age or older, who all had previous experiences of participating in a randomised controlled study [11]. In that study, they had all been screened as physically frail [12]. Contact details of potential participants were given to us by the researchers responsible for the study where they all had participated previously. In that study, the participants had been assessed repeatedly with quantitative interviews and physical tests in their own homes. However, what mattered was not what they had done in the study but that they all had some experience of participating in research, which was the point of departure for our project. Initial sampling criteria were set up aiming for diversity in age, sex, cognitive status [13], living situation, dependency in activities of daily living [14] and level of education. No exclusion criteria were set up because the aim was to invite a group of frail older people as diverse as possible to participate. This was in order to give them the opportunity to choose for themselves whether or not they perceived themselves of being capable of participating. A total of 31 potential participants were contacted at the hospital, or by phone if they had been discharged. Potential participants who did not answer the phone (*n* = 3) were sent a letter with information about the study and contact information. In total, 14 participants could not be reached (*n* = 7) or declined to participate (*n* = 7). Thus, 17 participants aged between 76 and 95 years were included, out of which eight were women and nine were men. Participants’ characteristics are shown in Table 1.

**Table 1:** Participants’ characteristics, *n* = 17

Variables	Value
Age, median (range)	85 (76–95)
Men, n (%)	9 (53)
Dependency in ADL, median (range)^a^	3 (1–9)
Higher education, n (%)^b^	6 (35)
Living alone, n (%)	*10 (59)*
Living in a nursing home, n (%)	3 (18)
MMT, median (range)^c^	28 (21–30)
Screened frailty factors, median (range)^d^	3 (2–4)
Decreased endurance, n (%)	16 (94)
Tired (last 3 months), n (%)	12 (71)
Fall tendency/fear of falling, n (%)	9 (53)
Help with grocery shopping, n (%)	9 (53)

^a^Degree of dependence in Activities of Daily Living (ADL) A higher score equals higher dependency [14]. Since continence was not considered an activity, nine was the max score

^b^Tertiary education (university or college)

^c^Mini-mental test [13]

^d^FRESH-Screen [12]

### Data collection and analysis

The data collection and analysis were conducted in parallel in accordance with grounded theory [10]. The data was collected through intensive interviewing using an interview guide with a number of question areas. The question areas focused on the participants’ experiences of involvement in research, the different parts of the research process and how their involvement in research could be influenced. For instance, they were asked about their thoughts on identifying research topics, designing studies, data collection and dissemination of findings in relation to research in general, based on their previous experiences. In line with Draucker et al. [15], the question areas were updated during the study process as a way of theoretical sampling to modify the data collection procedures to deepen the understanding of emerging concepts [15]. The interviews started with an open question: ‘Can you please tell me what it was that made you choose to participate in a research study?’ Follow-up questions were also used, for example ‘can you please give me an example of … ’ or ‘how do you mean when you say … ’ [10].

The interviews were conducted between February 2018 and March 2019 by three of the authors (IB (*n* = 15), EB (*n* = 1), SDI (*n* = 1)). The time and place for the interviews was chosen by the participants, and they all chose to be interviewed in their homes. Saturation was considered after 13 interviews, when the analyses did not yield any new findings on the questions studied. In the interviews that followed no new concepts emerged. The interviews lasted between 14 and 86 min, with a mean of 49 min. All the interviews were recorded digitally and transcribed verbatim. The qualitative data analysis software NVIVO pro 12 was used to organise and manage the interview data. The methods of initial coding, focused coding, constant comparison and memo-writing were used when analysing the data.

Firstly, the interviews were read/listened to as a whole, and thereafter an initial coding started using line-by-line coding. When coding line by line, the codes stayed close to the data and were mostly expressed in terms that attempted to capture actions and their connections to emotions and feelings. Thereafter the codes were organised into tentative categories to describe the participants’ different processes [10]. Secondly, focused coding was used to go through a larger amount of data, testing the meaning of the tentative categories. A constant comparison of codes/data within and between interviews using the tentative categories resulted in new categories with specific subcategories. The data was re-examined several times in an iterative process between the essence of the interviews and the meaning of the emerging categories and subcategories.

Field note memos were written soon after each interview, describing the context and setting. After the first examination of every interview, an analytical memo was written with a first impression. These memos were then used to identify different processes to examine further in coming interviews and the ongoing iterative process of analysis. Analytic memos were also written during the whole process of analysis to be able to compare codes, ideas and emerging categories both between and within the interviews [10]. In the results, quotes are used to give voice to the participants. All quotes are translated with breaks and repetitions removed for increased readability. All participants have been given pseudonyms. The numbers in brackets refer to which interview the citation is taken from.

### Ethics

This study followed the ethical principles of the World Medical Association’s declaration of Helsinki [16], and was approved by the Regional Ethics Committee at the University of Gothenburg (DNR T097–18). The participants were frail older people, some of them with cognitive impairment, who could have been considered a vulnerable group, and therefore they could have been excluded from participating. In the Helsinki declaration it is stated, however, that underrepresented groups should “be provided appropriate access to participation in research” [16] and this guided the inclusion of participants in the present study. To give the participants appropriate access, the language used in the information letter was adjusted by using short sentences and a large font size. Before the start of each interview, the participants had time to read the information and get it verbally from the interviewer. They were informed about the aim of the study, the voluntariness of participation, that they could terminate their involvement at any time without needing to give a reason, and that their involvement would not in any way affect their regular care or medical treatments. All interviews were conducted by healthcare professionals experienced in working with the target population and all participants were deemed competent to consent.

## Results

**Fig. 1:**
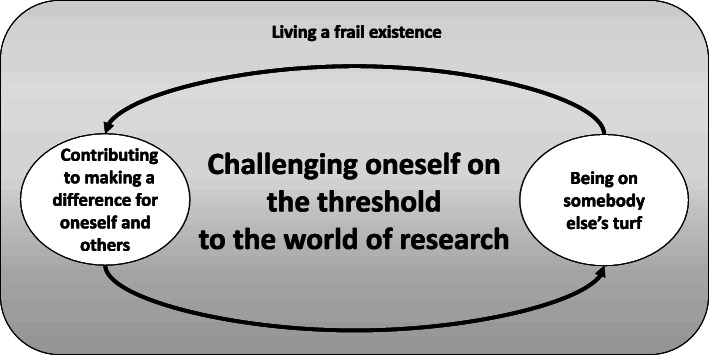
A figure to visualise the processes of the participants’ experiences of involvement in research and how the categories interact

From the perspective of the participants, the experience of being involved in research was understood as a process (see Fig. 1) that started in the category of *Contributing to making a difference for oneself and others*, which gave the participants the incentive to be involved in research and place themselves on the threshold to the world of research. This is described in the following subcategories: *Getting a break from everyday life*, *Wanting to address everyday problems, Wanting to enrich research with one’s historical perspective,* and *To do good for others.*

The participants’ involvement was influenced by them being in a context where their circumstances were influenced by *Living a frail existence.* Being involved in research meant that they as participants challenged themselves by placing themselves in the unusual position of *Being on somebody else’s turf*. This challenge in turn influenced whether they were content with their participation or whether they could find new incentives for involvement, which is described in the subcategories of *Doubting one’s role* and *Growing as a research participant.* This process is symbolised in the core category of *Challenging oneself on the threshold to the world of research*.

### Contributing to making a difference for oneself and others

When asked to participate in research, an opportunity of *Contributing to making a difference for oneself and others* was given, which worked as an incentive or motive for participation. By contributing to research, they were given the opportunity to influence their lives, both directly by being involved but also by using the occasion as a way of giving voice to their experiences. They were thus motivated both by selfish and more altruistic ideals. Four subcategories were identified; *Getting a break from everyday life, Wanting to address everyday problems, Wanting to enrich research with one’s historical perspective* and *To do good for others.*

#### Getting a break from everyday life

For the participants, one motive for getting involved in research was that they could get a break from everyday life and were given the opportunity for social exchange with a researcher. This meant that by being involved in research, they were given the opportunity to get time with another person, which was something they missed in their everyday lives. That it was a research activity mattered less than the interpersonal exchange taking place and that something out of the ordinary happened. As Majken described being visited by a researcher:

‘It’s fun, everything that happens is fun. You know, you’re so incredibly lonely when you are this old. So you welcome every break with open arms’ [Interview 4]

#### Wanting to address everyday problems

By being involved in research, the chance was given – or taken – to address problems close to everyday life. It was seen as an opportunity to talk about situations and events from everyday life or life in general that they experienced might be improved by more research. This could be self-perceived problems, things that had affected others nearby but also analyses of things that they had seen or come across in their surroundings. In dialogue with researchers, who the participants generally considered to be working with social development, a space was created for the frail older persons to express their concerns, perceived problems or other things that they wished that researchers would be able to contribute towards improving. One example of this was when Bengt said at the end of the interview:

‘I’ve got this thing … I don’t know if it has anything to do with this, but I am absolutely not happy with it, and there are many [who think] like me, and that’s accessibility when it comes to primary care’ [Interview 8]

#### Wanting to enrich research with one’s historical perspective

The participants saw themselves as an important group that should be given a greater opportunity to enrich research as they by virtue of their age have experiences and a historical perspective that no other group in society has. They described how they could make historical comparisons based both on their own personal experiences and experiences they shared with other older people. The participants experienced that society in general was bad at utilising older persons’ viewpoints and that by being involved in research, they would be able to enrich research and social development. For example, Allan described how older people’s involvement in research could make a difference:

‘Completely different questions and different perspectives so to speak. That would be appreciated on both sides I think.’ [Interview 5]

#### To do good for others

Involvement in research was described as an opportunity to be of use and make a positive contribution for others. This was because research was seen as something that contributes to progress and helps society to develop for the better. The participants also wanted to contribute on an interpersonal level by helping the researcher they had personal contact with to succeed with their project. The participants considered it to be their duty to participate and contribute if they could. The most important thing was not what the research was about but that it was them as persons who had been asked. Considering themselves to be representatives for their own group added to the motivation the participants felt to do good for research that could help the whole group of frail older people. One example can be found in how Ulla-Britt explains why she decided to be involved in research:

‘No, I’m thinking why wouldn’t one do it. If it helps someone in some way … Of course one should do it.’ [Interview 6]

### Living a frail existence

Living a frail existence was characterised by living with physical and social changes when everyday life is influenced by ageing. Ageing meant living with various physical limitations and illnesses that impacted on the conditions for how and where it was possible for them to be involved in research. As Göran who lived at a retirement home and used a wheelchair described the importance of the researcher making home visits:

‘I have very much been stuck here … Way too much … So I have gotten problems with my legs. I’m handicapped’ [Interview 10]

Ageing was described as a process with a changing social situation and diminishing social network as both family members and acquaintances their own age pass away. Living a frail existence was experienced as a change in the activities participants now were able to partake in compared to before. Märta, who only left her apartment once per quarter year, described this changing social network:

‘Because when all old friends are gone you get so lonely. And the kids, they have their own families and jobs after all so they can’t be with grandma all the time. It’s not possible’ [Interview 1]

There was both a carefulness and a longing to be involved in different types of activities because they had already stopped or been forced to cut back on activities in their lives. Having fewer activities and a diminished social network led to the experience that frail older people were outside of social development and that they were of lower status as a group.

### Being on someone else’s turf

When meeting with the researcher, the participants experienced inequality when they compared themselves to the high status and expertise that they experienced that the researcher stood for. Being involved in research was something new and unfamiliar that was not part of the participants’ everyday lives. The research topic played into the participants’ sense of insecurity in various ways depending on the extent to which they felt that the research came close to their own area of expertise, namely their everyday lives. Even when the researcher visited the participants in their everyday lives, they experienced it as though they were on somebody else’s turf. This was due to the fact that the visit was characterised by it being the visiting researcher who was in charge and controlled the conditions for how and when the participants were in the research process, which information they were given and when their role as research participants was over.

Two subcategories were identified; *Doubting one’s role* and *Growing as a research participant*. These were two different dynamic processes that to varying extents occurred simultaneously in the research participants and influenced how participants viewed their contribution to research and how they felt about further involvement in research.

#### Doubting one’s role as research participant

There was an uncertainty surrounding the expectations of what the role of a research participant would entail. The participants saw themselves as amateurs and were not sure about what they could contribute with as compared to other persons who they thought were better suited. Research was perceived as something that others do. When it was considered difficult to participate in research, the participants found comfort in the fact that a researcher had deemed them suitable to be involved in research. They dealt with their insecurities as research participants by doing as well as they could, at the same time as they tried to give the best possible answers based on what they expected that the researcher wanted to hear. Sune expressed his insecurities about what he would be able to contribute with:

Sune: No … No so I think that I don’t have much to contribute.

Interviewer: No.

Sune: No, I don’t you see.

Interviewer: To … To research or to the conversation?

Sune: To what you are after.

Interviewer: No. You say many interesting things Sune.

Sune: What?

Interviewer: You say many interesting things.

Sune: Yes. That can happen [Interview 14].

#### Growing in one’s role as research participant

In the process of research, as participants were affirmed by the researcher in that their experiences and thoughts were important and interesting, they also felt affirmed in being important to society and able to make a positive contribution. The respect that they felt they were given by the researcher influenced not only how the participants saw their involvement in the research process, but also how others in their surroundings were influenced in that they experienced the participants as being more respected as persons. One example of how participants thought they were being more respected by people in their surroundings can be found in Märta proudly showing a diploma that she received as a thank you for participating in another study:

“Märta: Yes, I don’t know but they [home health service staff] admire me differently. [they say] - Do you have a diploma, from the university.

Interviewer: Then they are impressed?

Märta: Yes, they are impressed. So am I. It’s a bit haughty to show this. They take a look, [and say] – what’s that? What have you been involved in?

Interviewer: You show the home health service staff?

Märta: Sure, they get to see it. I have it out, so they all can see it.” [Interview 1]

By growing as research participants, an interest and desire to be more involved could be evoked. The key to further involvement consisted of that researchers assessed them as valuable and of what would be demanded of them as participants.

### Challenging oneself on the threshold to the world of research

The core category of *Challenging oneself on the threshold to the world of research* symbolises the process that the participants went through when they reasoned with researchers based on their experiences of how they as persons could consider to be involved in a research project. The threshold to the world of research symbolises the distance they experienced between those who conduct research and those who participate in it. The way and the extent to which they wished to be involved in research ranged from standing on the threshold and being observed as an object on one end, to taking a step into the room of research and being involved as a partner on the other.

The participants’ motives for challenging themselves on the threshold to the world of research were influenced by their experiences of the frail lives they were living, but also by the experiences they gained from being involved on someone else’s turf and the incentives that this could give.

On the threshold, they were striving for equilibrium, and the challenge lay in balancing one’s own preconditions against the expectations growing out of the contrast of living a frail life and the unfamiliarity of being on someone else’s turf. This can be understood as a thought process where the participants sought to find their own role and form of involvement by assessing their capabilities in relation to what they themselves and the researchers wished for them to do.

## Discussion

The aim of this study was to explore frail older people’s experiences of involvement in research. The results showed that there seems to be variation in how frail older people want to and can be involved in research. This experience is shown in the core category *Challenging oneself on the threshold to the world of research* that is influenced by several different sub-processes that interact with one another. The study thereby contributes with knowledge on frail older people’s experiences of and reasoning about being involved in research. Earlier studies on frail older people’s view on involvement in research are scarce, if not non-existent. The little there is, is mostly written from the researcher’s perspective and is mostly anecdotal [17]. In a review by Brett et al. [18] on how involvement in research can impact on users, few studies are on older people in general, with most of them presumably being younger and healthier than the participants in our study [18].

A central finding of our study pertains to how frail older peoples’ experiences and reasoning about being involved in research is influenced by their experiences of power structures stemming from the contrast they experienced between their frail existence and research as something that is done on somebody else’s turf. In their frail existence, the participants had experiences of how physical changes influenced life and lead to an increasingly socially isolated existence. This experience shares similarities with how Sjöberg et al. [19] describe existential loneliness for frail older people. They describe how frail older people feel trapped in their frail and deteriorated bodies, which leads to isolation. How they feel abandoned, miss having somebody to share their everyday lives with, and a feeling of a lack of meaning when they do not feel connected to their surroundings and the rest of society [19]. Our results suggest that involvement in research could be a way of alleviating frail older peoples’ feelings of existential loneliness.

The fact that the participants experienced a difference in power between researchers and themselves as participants in our study could present a risk for participants experiencing their involvement as a symbolic representation (tokenism), that is to say that they experience that the researchers are not genuinely interested in what the participants contribute with. If the involvement of users in research is due to demands on the policy level, without a genuine interest on the researchers’ side to conduct research with users, their involvement runs the risk of being nothing more than what Buck et al. [20] describe as ‘ticking a political box’ [20].

The participants of our study had different motives or incentives for participating in research. These motives are similar to the proactive motives that Cox and McDonald [21] describe in their article on motives for research participation in health research. Proactive motives refer to when participants choose to participate based on their own volition [21]. The proactive motives of the participants in our study oscillate between favouring themselves (self-orientation) and others (social orientation). This was for instance the case in the subcategory of *Getting a break from everyday life,* which was a proactive way of fulfilling one’s own need of social interaction. Participants favouring themselves can, according to Cox and McDonald [21], lead to a feeling of increased empowerment. The feeling of empowerment may have been a part of how the participants in our study could *grow into the role of research participant* by way of feeling that they succeed in fulfilling their motives in the subcategories of *Wanting to address everyday problems* and *Wanting to enrich research with one’s historical perspective* [21]. Similar variation of motives for participation have been found in a study by Dahlin-Ivanoff et al. [22]. Even though the Dahlin-Ivanoff study was conducted on a younger and healthier sample, described motives for participation in research ranged from self-serving to altruistic ones. In our study, the participants highlighted the social dimension of participation. That this was not described by the participants in the study by Dahlin-Ivanoff et al. [22], which could be due to the studies’ different aims, or because the social dimension of participation might be more important for frail older people than the younger population in the Dahlin-Ivanoff study [22].

The participants found strength in receiving affirmation of being important and meaningful by researchers, who are a group that they perceive to be of high status. They were affirmed in that the historical perspective they could and wanted to contribute with also is of interest for society at large. The researcher’s attitude towards participants and the affirmation they received influenced the way in which they could *grow as research participants*. Dudley et al. [23] write about how the researcher’s attitude impacts on the experience of how users influence research. Those with a negative attitude towards user involvement experienced that the research had not been impacted, whereas those with a positive attitude found that the research had been influenced [23]. Our study participants’ experiences of how ageing had changed the conditions for what they can do and which social contexts they find themselves in may also explain why many may feel ill-suited to be research participants. At the same time, it also points to the importance of positive affirmation from a researcher that they are capable people when they are faced with the unfamiliar opportunity to be involved in research.

Our results emphasise the centrality of the relation between the researcher and the participants and the importance of the researcher’s attitude towards the participants’ capabilities, as the latter seems to be meaningful for how frail older people feel about the opportunity of being involved in research. Being able to cooperate and meet frail older people seem to be important skills that researchers must have in order for frail older people to be able to be involved in research. Our results point to the significance for there to be a space for variation in how frail older people can be involved based on their wishes and individual circumstances. One approach that allows for this variation can be found in Buck et al’s [19] description of a successful research cooperation between researchers and users, where the researcher needs to be both flexible and responsive. Being responsive and flexible means that users’ contribution is not pre-determined before the start of a project, but rather that it takes the form of a joined endeavour that entails a real sharing of power [19]. This may be contrasted to those who want to determine everything in advance, or are determined to do it together. One way of starting a research project between researchers and external actors is described in a case study by Barenfeld et al. [24]. Their project involved a process of finding a common ground to the problem of researchers and external actors coming from two different worlds where there is a lack of understanding for the other’s perspective, situation and view of the project. The process of finding a common ground was eased by clarifying the overarching expectations, using a shared language and trying to work on equal terms to counter hierarchical conceptions of power imbalance [24].

Challenges inherent in forming an equal relationship between researchers and participants emerged in our study and have also been described in previous research [25, 26]. One could argue that there are two different ways of diminishing this perceived inequality. One is that participants are offered education in order to thereby increase their knowledge about the process of research and thus come closer to the researcher’s high status [27]. The other way is to affirm the participants’ and researchers’ different areas of expertise, that it is precisely because they have not been schooled within academia that they can enrich research.

In the encounter between participants and researchers in our study, there was a power relation that was influenced by the fact that the two parts had different areas of expertise. Gaby et al. [28] propose that the power relation in the relationship between the researcher and participant is influenced by them having different expectations, interests, needs and feelings regarding research based on the different contexts they come from. One way for researchers to challenge the dominant ideologies and structures that tend to reduce research participants to objects is by way of adapting a person-centred approach towards the participants. A person-centred approach in research builds on a view that everyone involved are people who are relational beings and that it is in our relationships to other people that we can grow. From this view, this is what allows new knowledge to be formed in the meeting between researchers and participants [28].

Our study was built on showing a genuine interest and attention to the participants in order to create a space for communication through dialogue, where participants with their expertise would be able to make their voices heard and where our different perspectives would be seen as a strength in our collaboration. Similar to Gaby et al. [28], our results show that being involved in research can lead to a feeling of empowerment in the participants by way of allowing them to build their capacity in collaboration with researchers. For this knowledge that is shaped in the relation between researcher and participant to be able to be understood in its context, it is necessary for the researcher to adapt a critical reflexive approach. This requires the researchers themselves to be aware of their own position and status [28]. A person-centred approach in research can be that the researcher is responsive and flexible with regards to the participants’ circumstances, and contributes to optimising the participants’ influence on the research process based on this.

### Methodological limitations

According to Charmaz [10], the results of a study are dependent on its context, the situation, time, place and culture in which it is carried out. This study therefore needs to be understood within its specific context and the selection of participants. One limitation with this study is that the participants were discussing involvement in research without having any first-hand experience of it, or, for those who had experiences of it, discussing earlier experiences when their life situation was different. However, as the participants’ experiences were discussed in the context of collaboration with researchers, their experiences of participation in research as data sources with face to face contact with a researcher in the randomised controlled study they were recruited from, made them relevant for discussing involvement in research. Thus, even if our findings inform both quantitative and qualitative research, they are less applicable to research without face to face contact between frail older people and researchers, such as filling in surveys by themselves. Another limitation in our sample is that participants were recruited by people responsible for a larger population of participants in another study. That we ourselves were not entirely in charge of the process of inclusion may have influenced which people were asked to participate. For example, all our participants were Swedish-speaking. The experiences of involvement in research of frail older people who do not speak Swedish is thus something our results do not address.

One strength in our population is that we were able to include people with a range of cognitive abilities. That people with cognitive impairment are excluded from research is still common in geriatric research, often without explanation of why or any discussion on how it influences the results’ representativeness of older people as a group, where cognitive impairment is common. It seems as though dementia or cognitive impairment are regarded as impacting on the possibility to participate in research [7], instead of considering all participants to be individual persons with different capabilities. In our material there are for example interviews with people with low MMT, and in spite of, or precisely because of this, these interviews could contribute to our results with important insights and experiences.

Not having the strength, will or capacity to be interviewed for a certain length of time should not be what matters for whether or not one is considered suitable for participation, but rather it is what is said that matters and that is not possible to know before the interview has been conducted. An interview lasting 14 min can contain more important pieces of a puzzle than an interview that lasts several hours. In this study, no interview had to be terminated because a participant felt tired; rather, there was a desire for more conversation and social exchange. There may be a risk when the researcher starts the interview with the view that the other is a vulnerable person that one needs to be especially careful with.

## Conclusions

In conclusion, this study contributes with an understanding of how frail older people can and want to be involved in research, and that frail older people have a varied capacity to be involved. But in what way and how is difficult to know before they have been involved in the process of research. In collaboration with researchers, frail older people are given the chance to assess what they want to and are able to do. Our results advocate that it is problematic to exclude frail older people a priori and that there is potential for new perspectives and knowledge to be shaped in the encounter and in the relationship between the researcher and the frail older person.

In the research relationship between the frail older person and the researcher, there are opportunities, but also obstacles based on the different worlds both come from. In research without user involvement, there are needs, perspectives and resources that run the risk of being left out. For research to be able to cater to frail older people’s needs of health services, their voices need to be heard and taken into consideration. Otherwise, there is a risk that health services will continue to treat the group of frail older people based on the wishes and perspectives of other groups.

## Data Availability

This is a qualitative study and the datasets generated and analysed during the current study are not publicly available due to the information provided to the participants when obtaining their informed consent, stating that all attempts would be made to maintain confidentiality. De-identified data are, however, available upon reasonable request to enable review, and will be stored for 10 years at the University of Gothenburg. All data are covered by the Public Access to Information and Secrecy act (offentlighets- och sekretesslagen) and a confidentiality assessment (sekretessprövning) will be performed at each individual request. Permission from University of Gothenburg, the Institute of Neuroscience and Physiology, has to be obtained before data can be accessed.

## Abbreviations

ADL: Activities of daily living
MMT: Minimal Mental Test
